# Genome-scale metabolic analysis of *Clostridium thermocellum *for bioethanol production

**DOI:** 10.1186/1752-0509-4-31

**Published:** 2010-03-22

**Authors:** Seth B Roberts, Christopher M Gowen, J Paul Brooks, Stephen S Fong

**Affiliations:** 1 Department of Chemical and Life Science Engineering, Virginia Commonwealth University, Richmond, VA, 23284, USA; 2 Department of Statistical Sciences and Operations Research, Virginia Commonwealth University, Richmond, VA 23284, USA; 3 Center for the Study of Biological Complexity, Virginia Commonwealth University, Richmond, VA 23284, USA

## Abstract

**Background:**

Microorganisms possess diverse metabolic capabilities that can potentially be leveraged for efficient production of biofuels. *Clostridium thermocellum *(ATCC 27405) is a thermophilic anaerobe that is both cellulolytic and ethanologenic, meaning that it can directly use the plant sugar, cellulose, and biochemically convert it to ethanol. A major challenge in using microorganisms for chemical production is the need to modify the organism to increase production efficiency. The process of properly engineering an organism is typically arduous.

**Results:**

Here we present a genome-scale model of *C. thermocellum *metabolism, *i*SR432, for the purpose of establishing a computational tool to study the metabolic network of *C. thermocellum *and facilitate efforts to engineer *C. thermocellum *for biofuel production. The model consists of 577 reactions involving 525 intracellular metabolites, 432 genes, and a proteomic-based representation of a cellulosome. The process of constructing this metabolic model led to suggested annotation refinements for 27 genes and identification of areas of metabolism requiring further study. The accuracy of the *i*SR432 model was tested using experimental growth and by-product secretion data for growth on cellobiose and fructose. Analysis using this model captures the relationship between the reduction-oxidation state of the cell and ethanol secretion and allowed for prediction of gene deletions and environmental conditions that would increase ethanol production.

**Conclusions:**

By incorporating genomic sequence data, network topology, and experimental measurements of enzyme activities and metabolite fluxes, we have generated a model that is reasonably accurate at predicting the cellular phenotype of *C. thermocellum *and establish a strong foundation for rational strain design. In addition, we are able to draw some important conclusions regarding the underlying metabolic mechanisms for observed behaviors of *C. thermocellum *and highlight remaining gaps in the existing genome annotations.

## Background

Constraint-based modeling is a rapidly expanding approach to studying biological systems on the genome scale. Models for a range of different organisms with varying metabolic capabilities have been published over the last ten years [1-4]. Each of these models is fundamentally defined by a list of mass-balanced, and possibly, charge-balanced reactions. Thermodynamic [5,6] and other types of constraints can also be incorporated to these models to provide added detail. By means of the quasi-steady state assumption, i.e., that metabolite concentrations are constant over short time scales, the reaction list can be used to define a space of possible steady state behaviors for the metabolic network. This solution space can then be probed by a growing number of methods to obtain specific predictions of the organism's behavior [4,7-9]. The most commonly employed method is flux balance analysis (FBA). In FBA, a metabolic objective is specified (usually biomass production) and linear programming is used to identify a single point in the space of possible steady-state metabolic states that maximizes this objective. Of the commonly used objectives, evidence suggests that biomass optimization is most consistent with experimentally observed flux distributions in carbon-limited cells grown in batch culture [10-12].

The constraint-based approach to metabolic modeling fundamentally represents a functional, in-context method for studying cellular metabolism. Through various analyses, such as FBA, predictions of cellular behavior can be made to test or confirm our current state of knowledge about a specific organism's metabolic network. Not only does this give us a computational means of predicting function from genomic information, but the process of building these models represents an added level of functional annotation within the context of an overall network [13]. Both the predictive capabilities and the potential improvements to genome annotation are useful particularly when studying relatively poorly characterized organisms. Thus, constraint-based models provide a framework for assessing the functional biochemical network of an organism that can be used to study fundamental metabolic functions.

A variety of cellulolytic microorganisms exist that have the potential to utilize cellulose for biofuel production, but these organisms are typically poorly characterized. The application of constraint-based metabolic modeling to cellulolytic organisms may help increase our understanding of how these organisms function and lead to improvements in bio-based biofuel production. The utilization of cellulose for the production of ethanol or other fuels has recently been highlighted [14-16] as an important objective and a necessary step in order to sustainably harvest a renewable energy source that can reduce dependence on petroleum derived fuels. Currently, ethanol is derived from biomass using at least two distinct steps: 1) enzymatic saccharification of biomass, and 2) fermentation. The cost of enzyme production and treatment has significantly impeded more widespread use of this technology [17]. *Clostridium thermocellum *has generated a great deal of interest, because it both hydrolyzes cellulose and produces ethanol as a fermentation product and therefore has potential to be a model organism for consolidated bioprocessing (CBP) that eliminates the separate saccharification step [18]. If such a process could be industrialized, it would represent an important new and efficient method of bioethanol production.

*C. thermocellum *produces a number of industrially important fermentation products in addition to ethanol, including acetic acid, formic acid, and hydrogen (H_2_). Thus, *C. thermocellum *is a potentially important candidate for metabolic engineering, to divert energy and carbon flow toward desired fermentation products. Recent investigations of a related organism, *Clostridium acetobutylicum *[19-21], suggest that the metabolic networks of these Clostridia may operate in unconventional ways. This implies that many of the necessary manipulations for optimal strain design could be non-intuitive. Genome-scale constraint-based models have proven to be very powerful tools for metabolic engineering, because they provide global, integrated views of metabolism, allowing both discovery and assessment of possible manipulations [11,12,17,22]. Thus, there is a strong rationale for developing a genome-scale metabolic model for *C. thermocellum *from an application perspective.

In this paper, we present a genome-scale model of *C. thermocellum *(ATCC 27405) consisting of 577 reactions involving 525 distinct metabolites, 73 membrane transport reactions, and 432 genes (19.1% of all *C. thermocellum *genes with function prediction). The reaction list was compiled based on the genome annotations available in the UniProt [23], Kyoto Encyclopedia of Genes and Genomes (KEGG) [24-26], and integrated microbial genomes (IMG) [27] databases along with published phenotypic information. The constructed model includes a novel proteomic-based cell structure (cellulosome) and was analyzed for 1) accuracy compared to experimental results, 2) effects of genetic and environmental changes, and 3) metabolic differences from other related organisms.

## Results

In this study, the cellulolytic ethanologen *C. thermocellum *was computationally analyzed by integrating genomic, biochemical, and physiological information. The main result of this work was the development of a genome-scale metabolic model of *C. thermocellum *that includes a unique model representation of a cellulosome, a major functional unit in cellulose hydrolysis that accounts for a high percentage of the total protein content in *C. thermocellum*. The accuracy of the constructed model was determined by comparing the results of model simulations to experimental results for growth on cellobiose and fructose from two different independent studies. FBA was used to study the range of ethanol production capabilities of both wild-type and gene deletion strains of *C. thermocellum *in different chemical environments. Metabolic network comparisons were also conducted between *C. thermocellum *and a related butanol-producing species *C. acetobutylicum *and between *C. thermocellum *and the model ethanologen, *Saccharomyces cerevisiae*. All computational and experimental work was based upon the wild-type strain of *C. thermocellum *(ATCC 27405). The final reaction and metabolite list is available in Excel format (Additional file 1), and the complete model is available in tab-delimited text format (Additional file 2) and SBML format (Additional file 3).

### Model Description

Based upon genomic and available physiologic evidence, a genome-scale constraint-based metabolic model for *C. thermocellum*, hereafter denoted *i*SR432, was developed. *i*SR432 contains 577 reactions representing the function of 432 genes (see Table 1). Of the 577 reactions, 73 represent transport processes from the extracellular space to the cytoplasm, or vice-versa. Four hundred sixty-three of the reactions are associated with genes. Of the reactions that are not associated with genes, 60 are intracellular and 54 are transport reactions. The metabolic subsystems with the highest number of reactions missing gene assignments are transporters and cell envelope biosynthesis. The fact that many transporters are not assigned genes is not surprising in light of the fact that these processes are generally not as well-characterized as metabolic reactions due to the potential multi-functionality of transport mechanisms. The absence of gene assignments for many of the reactions in cell envelope biosynthesis is mainly due to the fact that many of these are lumped reactions, representing several sequential metabolic transformations (see Methods section). There are 525 distinct metabolites included, counting extracellular and intracellular forms of the same species as one.

**Table 1:** Overview of four genome-scale constraint-based models related to ethanol or butanol production.

	*C. thermocellum iSR432*	***C. acetobutylicum ***[20]	***C. acetobutylicum CacMBEL502 ***[21]	***S. cerevisiae iND750 ***[3]
Genome size	3.8 Mb	4.1 Mb	4.1 Mb	12.2 Mb
ORFs	3307	4017	4017	6276
Included genes	432	458	432	750
Enzyme complexes	72	n/a^a^	36	86
Isozyme cases	70	n/a	n/a	145
Reactions (excluding exchanges)	577	552	502	1150
Transport	73	80	71	308
Gene associated	463	414	431	810
Non-gene associated intracellular	60	119	n/a	123
Non-gene associated transports	54	19	n/a	216
Distinct metabolites	525	488	479	646

^a^n/a -- data not available

**Figure 1:**
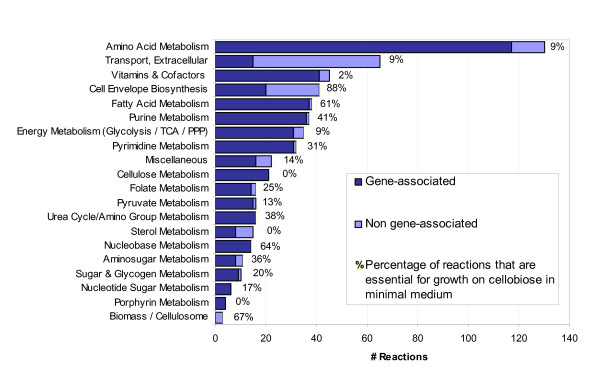
**Distribution of reactions in *C. thermocellum i*SR432 model by functional category**. Each reaction in the model is assigned to a single functional category. The length of each bar indicates the total number of reactions falling into each category, and the dark- and light-blue portions indicate the number of reactions that are currently mapped to *C. thermocellum *open reading frames (gene-associated) or not (non gene-associated), respectively. The bar labels indicate the percentage of reactions in each category which are predicted to be essential for growth on cellobiose in MJ minimal medium [28].

The breakdown of *i*SR432 by functional categories is represented in Figure 1. Amino acid metabolism forms the largest of these, with 126 reactions. Experimentally, *C. thermocellum *appears to have the capacity to synthesize all 20 amino acids [28], and *i*SR432 reflects this. Other large groups of reactions include subsystems related to carbon source processing (glycolysis, pentose phosphate pathway, pyruvate metabolism, and the citric acid cycle) and cell envelope biosynthesis (including reactions related to phospholipid, peptidoglycan, and teichoic acid synthesis). The model includes reactions relating to the synthesis of a variety of vitamins and cofactors, including biotin, NAD, pantothenate, and riboflavin.

#### Initial reconstruction

The initial reconstruction of *C. thermocellum *metabolism was assembled based on the existing annotations (see Methods) and using a database of biochemical reactions compiled from previously published genome-scale constraint-based models, resulting in an initial set of approximately 400 reactions. This initial version was incomplete in several respects, including gaps in critical metabolic subsystems and the inability to synthesize metabolites known to be crucial (e.g., phospholipids). This initial version was then supplemented with reactions representing processes known to be crucial, but missing in this first version. Some of these reactions were missing because they represent either processes specific to *C. thermocellum*, or processes that must be represented by manually constructed, lumped reactions. Examples include cellulosome production, cellulose and chitin breakdown, fatty acid synthesis, phospholipid synthesis, teichoic acid and peptidoglycan synthesis, steroid metabolism, and various transport processes known to occur in *C. thermocellum*. We also added missing transport reactions for important inputs (e.g., water) and known metabolic fermentation products (e.g., formate). This second model version resulted in the addition of about 150 additional reactions.

#### Gap filling

After the aforementioned changes, the resulting second version was still incomplete due primarily to gaps in critical metabolic subsystems. These were addressed by manual curation of the individual subsystems, using temporary exchange reactions (temporary artificial transport mechanisms used to isolate a subsystem of pathways) to identify missing components. For example, in glycolysis, there was no pyruvate kinase included in the early versions of the model, because none of the existing genome annotations included a gene with this annotated function. However, upon inspection of the glycolysis pathway, this gap became self-evident. Because essentially all of the surrounding reactions (i.e., reactions leading up to and away from the missing pyruvate kinase reaction) were present and because pyruvate kinase activity has been experimentally observed in *C. thermocellum *[29], we decided to include this reaction even though there was initially no genetic evidence. In an attempt to discover genes of *C. thermocellum *that might code for a missing enzyme, we used BLASTP similarity searches between the translated set of *C. thermocellum *genes and enzymes from public databases with the annotation of interest. For example, for pyruvate kinase (EC 2.7.1.40) all sequences from UNIPROT annotated with this EC number (2.7.1.40) were downloaded. Using BLASTP, we identified reciprocal best hits (RBH) between the set of genes in *C. thermocellum *and the set of genes from UNIPROT annotated to EC 2.7.1.40, using an e-value cutoff of 10^-5^. The complete list of reciprocal best hits can be found in Additional file 1 on the sheet 'final RBH.' There were generally several RBH, and from these we selected a candidate *C. thermocellum *gene that could plausibly perform the function in question. Similar procedures were repeated for all other model subsystems, until we were able to generate a positive flux on the biomass reaction when analyzing the model by FBA. Gaps that were filled using reactions that were not associated with direct genetic evidence were noted. The results of this analysis (shown in Table 2) are 27 genes that likely should have annotations added/modified for *C. thermocellum*.

**Table 2:** Possible new annotations for *C. thermocellum *ORFs based on identified metabolic gaps

Missing EC number	Enzyme name	Possible Cth ORF	Current annotation	Reciprocal best hit^a^	E value^b^
2.7.1.107	diacylglycerol kinase	Cthe_3168	hypothetical protein	A0R923	7.00E-35

3.2.1.52	Hexosaminidase	Cthe_0787	isoleucyl-tRNA synthetase	A4N847	0

2.7.7.39	CDP-glycerol pyrophosphorylase	Cthe_1276	pantetheine-phosphate adenylyltransferase	A1SHB9	3.00E-07

2.7.8.8	phosphatidylserine synthase	Cthe_0158	Ribonuclease	A9JBA9	1.00E-68

4.1.1.65	phosphatidylserine decarboxylase	Cthe_0505	formate acetyltransferase	A4NBN7	0

1.3.99.1	succinate dehydrogenase	Cthe_2355	L-aspartate oxidase	Q97W79	1.00E-94

1.3.1.6	NADH-fumarate reductase	Cthe_2355	L-aspartate oxidase	B0VG44	5.00E-98

1.3.5.1	succinate dehydrogenase	Cthe_2355	L-aspartate oxidase	A4YEK0	2.00E-85

6.2.1.5	succinate--CoA ligase	Cthe_1907	amino acid adenylation domain	A3P3B3	3.00E-40

6.4.1.2	acetyl-CoA carboxylase	Cthe_0699	carboxyl transferase	A0RY61	8.00E-169

6.3.4.14	biotin carboxylase	Cthe_0949	carbamoyl-phosphate synthase, large subunit	B2J980	0

4.1.3.38	aminodeoxychorismate lyase	Cthe_0026	queuosine biosynthesis protein	Q03L66	3.00E-54

3.1.3.1	alkaline phosphatase	Cthe_2965	binding-protein-dependent transport systems inner membrane component	B0USD4	1.00E-59

2.6.1.2	alanine transaminase	Cthe_0755	aminotransferase, class I and II	Q7LYW0	3.00E-66

2.6.1.51	serine--pyruvate transaminase	Cthe_0265	aminotransferase, class V	B4BE13	0

2.7.1.39	homoserine kinase	Cthe_0397	ABC transporter related protein	A5 M0U7	3.00E-140

3.1.3.3	phosphoserine phosphatase	Cthe_0256	histidine kinase	A9G173	3.00E-54

2.7.1.40	pyruvate kinase	Cthe_1955	RNA binding S1	A5LC67	0

1.2.2.1	formate dehydrogenase	Cthe_0199	4Fe-4S ferredoxin, iron-sulfur binding	Q2LVY6	9.00E-11

1.7.99.4	nitrate reductase	Cthe_0200	FAD-dependent pyridine nucleotide-disulphide oxidoreductase	Q11VH4	4.00E-24

2.2.1.2	transaldolase	Cthe_0217	Glucose-6-phosphate isomerase	Q2S6E8	1.00E-22

6.3.4.1	GMP synthase	Cthe_0375	GMP synthase, large subunit	A2C5P2	4.00E-176

1.2.1.2	formate dehydrogenase	Cthe_0341	NADH dehydrogenase (quinone)	B5IPC7	6.00E-126

5.3.3.2	isopentenyl-diphosphate Delta-isomerase	Cthe_1022	Glycerol-3-phosphate dehydrogenase	A8VXT5	2.00E-94

2.5.1.29	farnesyltranstransferase	Cthe_0831	Polyprenyl synthetase	B2J443	4.00E-69

2.5.1.33	trans-pentaprenyltranstransferase	Cthe_0564	Trans-hexaprenyltranstransferase	Q6KZR8	3.00E-25

3.2.1.108	lactase	Cthe_0212	Beta-glucosidase	P09848	3.00E-89

3.5.1.19	nicotinamidase	Cthe_1178	isochorismatase hydrolase	Q6F6U3	6.00E-08

1.2.4.4	branched chain keto acid dehydrogenase	Cthe_0547	periplasmic solute binding protein	A8VXE7	2.00E-27

^a^UniProt accession numbers

^b^E value based on reciprocal best hit against *C. thermocellum *gene

#### Citric acid cycle and the fate of succinate

We noted during model curation that the citric acid cycle was complete except for two reactions, namely succinate dehydrogenase and succinate:CoA ligase. There was no clear genetic evidence for these genes, although similarity searches produced some probable reciprocal best hits (see Table 2). Due to the importance of these genes and their potential effect on metabolism, we conducted experimental assays for succinate dehydrogenase (SDH) activity in *C. thermocellum*. Using a colorimetric assay that can detect the activities of various dehydrogenase enzymes, we measured succinate dehydrogenase and lactate dehydrogenase (LDH) activity in both *E. coli *(control) and *C. thermocellum *(LDH as a control). We were able to detect LDH activity in both *C. thermocellum *and E. coli as well as SDH activity in *E. coli *(data not shown). We were unable to detect SDH activity in *C. thermocellum*. Based on the absence of genetic and experimental biochemical evidence for this reaction, we elected to exclude the succinate dehydrogenase reaction from *i*SR432. We did, however, add a reaction for succinate:CoA ligase, based on the fact that succinate has been reported as a metabolic fermentation product of *C. thermocellum *[30,31] and genetic evidence from our similarity searches.

### Comparison of model results to experimental results

**Figure 2:**
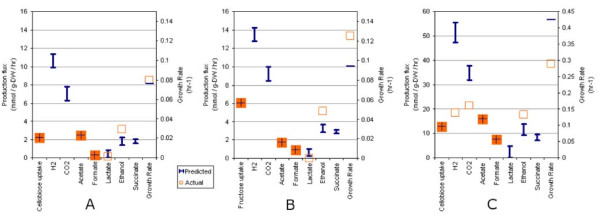
**Comparison of model predictions to experimental observations**. *C. thermocellum iSR432 *was used to simulate growth in multiple conditions. Actual and predicted reaction flux rates are shown, and predicted fermentation product production rates are shown as ranges as determined by flux variability analysis (see Methods). For each simulation, the boundary fluxes for cellobiose, acetate, and formate were constrained to match the measured fluxes during (A) chemostat growth on cellobiose and (B) fructose[32], and (C) batch growth on cellobiose[33].

To test the predictions of *i*SR432, we simulated growth of *C. thermocellum *by applying FBA, assuming minimal media conditions with one of two possible carbon sources (cellobiose or fructose). We then compared model predictions to experimentally observed growth rates and fermentation product secretion profiles of *C. thermocellum *grown in either continuous [32] or batch [33] culture. In addition to the carbon source, the *in silico *minimal medium used for simulations contained water (h2o), ammonia (nh4), sulfate (so4), phosphate (pi), calcium (ca2), ferrous iron (fe3), hydrogen sulfide (h2s), potassium (k), magnesium (mg2), pantothenate (pnto-r), and nicotinate D-ribonucleotide (nmn). In each of the three simulation conditions (continuous-cellobiose, continuous-fructose, and batch-cellobiose), we applied progressively more experimentally determined constraints [32] associated with by-product secretion rates to determine how closely the computational results could match the experimental results given the possibility of alternate optimal solutions [34]. Figure 2 shows the simulation results (given as the possible range of reaction fluxes) for each growth condition when exchange rates for the carbon sources, acetate, and formate were constrained to match experimental observations. Detailed results for these simulations are also provided in Additional file 1.

#### Continuous Growth on Cellobiose

Fermentation experiments in which *C. thermocellum *grew in continuous culture and consumed cellobiose at 2.2472 mmol gDW^-1 ^hr^-1 ^[32] were the basis for testing model predictions of continuous growth on cellobiose. The experimental observations for growth rate, cellobiose uptake and major metabolic byproduct production rates can be found along with selected model predictions in Figure 2(A). In this case, the experimentally measured growth rate was 0.08 hr^-1 ^[32]. Based on experimental evidence that growth on cellobiose is associated with less cellulase activity[35], we set the cellulosome production requirement for these simulations to zero. Applying one constraint, for cellobiose uptake flux, gave a predicted growth rate of ~.094 hr^-1^. Applying an additional constraint for acetate production resulted in a predicted growth rate of ~0.076 hr^-1 ^and greatly improved the prediction for ethanol production. Fermentation products predicted by the model, but for which we had no experimental measurements, included CO2, and H2. To consider the effect of our computational representation of the cellulosome on growth predictions, we restored the cellulosome production requirement back into the objective function. This resulted in a decrease of ~4-17% in predicted growth rates, depending on which constraints were applied. This decrease in the predicted growth rate represents the metabolic impact (burden) of producing cellulosomes in *C. thermocellum*.

#### Continuous Growth on Fructose

In the case of continuous growth on fructose, the experimentally measured growth rate was 0.125 hr^-1^[32]. Experimental observations and major metabolic fermentation product productions rates for these conditions are in Figure 2(B). Based on experimental evidence that growth on fructose is associated with more cellulase activity [35], cellulosome production was required as part of these simulations. Applying a single constraint for fructose uptake flux resulted in a predicted growth rate of 0.123 hr^-1^. Applying one further constraint for acetate production resulted in a predicted growth rate of 0.095 hr^-1^. Applying constraints for acetate, lactate, ethanol, and formate production resulted in a predicted growth rate of 0.090 hr^-1^. Removing the cellulosome production requirements resulted in an increase of ~4-8% in growth rates, depending on which constraints were applied.

#### Batch Growth on Cellobiose

Fermentation experiments in which *C. thermocellum *grew in batch culture and consumed cellobiose at 12.8 mmol gDW^-1 ^hr^-1 ^[33] were the basis for testing model predictions of batch growth on cellobiose. The experimental observations for growth rate, cellobiose uptake and major metabolic product secretion rates can be found along with selected model predictions in Figure 2(C). When only the cellobiose uptake rate is specified, *i*SR432 predicts a maximum growth rate of 0.518 hr^-1^, which correlates with high production of H_2_, CO_2_, and acetate and very low production of all other major metabolites. As other constraints are added to match observed fluxes, the maximum growth rate, as in other cases, tends to drop, and carbon flux is redistributed to other metabolites. When constraints for acetate, formate, ethanol, H_2_, and CO_2 _are added, flux balance analysis predicts a maximum growth rate of 0.387 hr^-1 ^and very low or absent production of lactate and succinate Furthermore, when succinate and lactate production are also constrained, excess carbon is relieved by an increase in the production of aspartate to 8.48 mmol gDW^-1 ^hr^-1 ^(see Additional file 1). With these constraints, the maximum predicted growth rate is 0.380 hr^-1^, and as additional constraints are added to reduce all amino acid export fluxes to zero (not shown), the maximum growth rate gradually drops to below the observed value until *in silico *growth is impossible with no additional amino acid production.

### Gene Deletions: essentiality and effects on ethanol secretion

We conducted comprehensive *in silico *single gene deletions with *i*SR432, using cellobiose as a carbon source and the other minimal media components (as described above), and constraining cellobiose uptake to its experimentally observed value for batch growth (12.8 mmol gDW^-1 ^hr^-1^, shown in Figure 2). Gene essentiality results are shown in Figure 1. In the case of growth on cellobiose, we found that 208 (36%) of *C. thermocellum *genes included in *i*SR432 were predicted to be essential. We also examined which subsystems of *i*SR432 contained the highest percentage of essential reactions ('vulnerable subsystems'). Among the most vulnerable subsystems are the cell envelope biosynthesis, nucleobase metabolism, and fatty acid metabolism.

**Figure 3:**
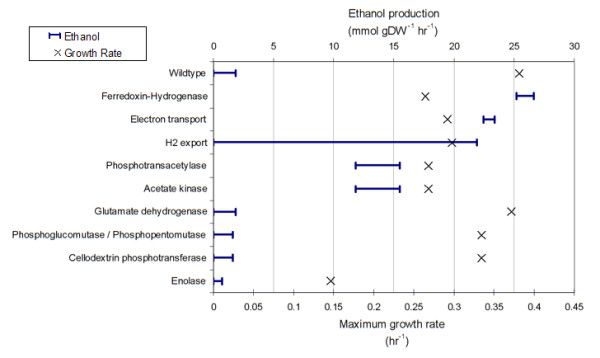
**Single gene deletions associated with increased ethanol production**. The predicted maximum growth rate (X) is shown for each simulated deletion scenario during growth on a cellobiose minimal medium with a measured cellobiose uptake rate of 12.8 mmol gDW^-1 ^hr^-1^. Also shown is the range of ethanol production that can be achieved at the maximum growth rate See Additional file 1 for details about the genes deleted and the affected reactions.

For each gene deletion predicted to be non-lethal, we conducted flux variability analysis to determine the effect of the deletion on the lower and upper bounds for ethanol secretion. Results are shown in Figure 3. There were nine single gene deletions (Cthe_1028, Cthe_1029, Cthe_2430, Cthe_2431, Cthe_2432, Cthe_2433, Cthe_2434, Cthe_2435, and Cthe_3003), associated with seven distinct model reactions, that were predicted to increase the upper bound on ethanol secretion relative to the wild-type ethanol secretion capabilities. Each of these was also predicted to increase the lower bound on ethanol secretion indicating that a deletion of one of these genes should force an increase in ethanol production. Deletion of gene Cthe_3003 (coding for R_FDXHASE or Ferredoxin hydrogenase 1.12.7.2) was predicted to result in the greatest change that resulted in a ~15 fold increase in maximum ethanol secretion and a concomitant 30% decrease in biomass production, when compared to ethanol production at optimal growth rate of the wild-type strain. Genes predicted to increase ethanol secretion upon deletion were either involved in reactions affecting reduction-oxidation balance or in reactions producing acetate.

### Tradeoff of H_2 _and ethanol production

**Figure 4:**
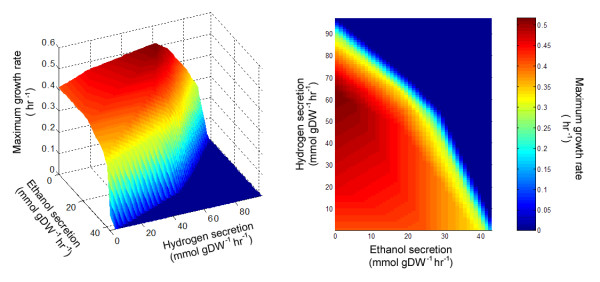
**The effect of gaseous hydrogen secretion on the fermentation product secretion profile of *C. thermocellum i*SR432**. The escape flux of H_2 _was varied incrementally across the viable range, and FBA was used to determine the maximum growth rate and the concomitant fermentation product escape fluxes, given a cellobiose uptake rate of 2.2472 mmol gDW^-1 ^hr^-1^. Flux variability analysis was used to determine the full range of ethanol flux (dotted orange lines) possible at each test value.

In examining the trends shown in Figure 2, we noted a general tradeoff between the flux on H_2 _production and ethanol production. A number of studies [36-39] have suggested that by thermodynamically restricting hydrogen escape, it is possible to increase the production of ethanol by *C. thermocellum*. To investigate this phenomenon, we tested the relationship between H_2 _escape and the production of other fermentation products during batch growth on cellobiose. Over the entire ranges of hydrogen and ethanol production that would allow *in silico *growth, we constrained H_2 _and ethanol escape and used FBA to determine the maximum growth rate. The results are displayed in Figure 4 and indicate that at the highest possible growth rate, ethanol production drops to zero while H_2 _production yield is

If hydrogen or ethanol secretion are constrained away from this global maximum, the *in silico *organism is forced to utilize less efficient pathways to varying degrees. For example, when ethanol and hydrogen escape fluxes are both forced to zero, the carbon flux to ethanol is rerouted at acetyl-CoA to acetate production, and since H_2 _can no longer act as an electron acceptor for reduced ferredoxin produced by pyruvate:ferredoxin oxidoreductase (R_POR2_i; 1.2.7.1), acetyl-CoA is instead produced by pyruvate formate lyase (R_PFL; 2.3.1.54), resulting in a drastic increase in formate export (see Additional file 1, sheets 'h2_etoh_flux distributions' and 'h2_etoh_nonzero exchange fluxes' for the complete simulation results).

### Comparative Analysis of metabolisms - compare to *C. acetobutylicum *and *S. cerevisiae*

**Figure 5:**
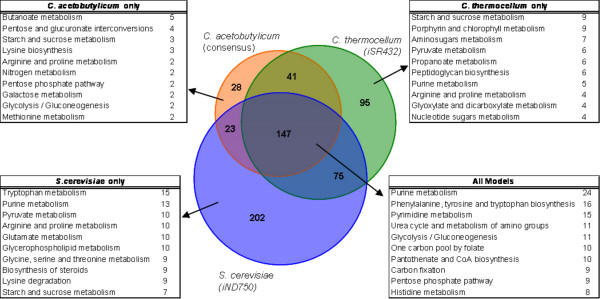
**Comparison of model content for three ethanologenic organisms**. *C. thermocellum i*SR432, *C. acetobutylicum *consensus [CacMBEL502 [21] and [20], and *Saccharomyces cerevisiae *iND750 were found to exclusively represent 95, 28, and 202 EC numbers, respectively, and 147 EC numbers were shared among all three. The EC numbers were mapped to pathway names using the Kyoto Encyclopedia of Genes and Genomes (KEGG) database, and the top ten most frequently occurring pathways for each of the exclusive lists and the combined list are shown along with the count of occurrences for each pathway.

One consequence of constructing genome-scale models is the ability to obtain a comprehensive overview of an organism's metabolic network. Using our reconstructed network for *C. thermocellum*, we sought to computationally compare the metabolic network of *C. thermocellum *to a related, but butanol-producing Clostridia species (*C. acetobutylicum*) and a different ethanologenic microorganism (*S. cerevisiae*). We conducted our analysis by comparing the content of the metabolic models for these three organisms, shown in Figure 5. As described in Methods, we represented reaction content of the models by EC numbers. The EC numbers unique to *C. thermocellum *are commonly associated to pathways such as Starch and Sucrose Metabolism (especially due to enzymes involved in cellulose metabolism) and Porphyrin Metabolism (several enzymes related to Vitamin B12 metabolism and production of various porphyrinogens). EC numbers unique to *C. acetobutylicum *are commonly associated to pathways such as Butanoate Metabolism (CAC is the only one of the three organisms that natively produces butanol) and Pentose and Glucuronate Interconversions. EC numbers unique to *S. cerevisiae *are commonly associated to pathways such as Tryptophan Metabolism and Purine Metabolism. Finally, the EC numbers shared by all three models are commonly associated to pathways such as Purine and Pyrimidine Metabolism and aromatic amino acid biosynthesis. No doubt, especially in the latter set, one reason that these particular pathways are well represented is because they are associated with many EC numbers, i.e., they are "big" subsystems. As expected, core pathways of metabolism, such as glycolysis and the pentose phosphate pathway, are also among the most commonly represented in the set of EC numbers common to all three organisms.

### Simulation of alternative media formulations

**Figure 6:**
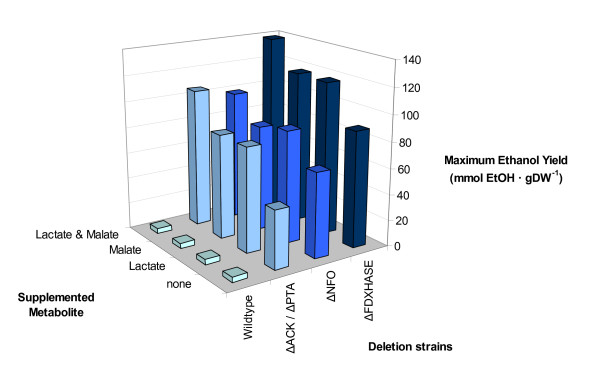
**Alternative media formulations for single-reaction deletion strains**. *C. thermocellum iSR432 *was used to simulate the addition of various potential media components individually and in pairs (see Methods for details). The maximum possible ethanol yield per biomass is shown for four deletion strains during simulated growth on cellobiose and supplemented alternative carbon sources.

Media formulations and growth conditions can significantly affect the fermentation characteristics of any microorganism. In light of this, we used *i*SR432 to make context-dependent predictions of growth and fermentation product secretion profiles. Specifically, the additions of 35 metabolites were systematically simulated, both individually and in combinations of two, and flux variability analysis was performed to determine the resulting maximum growth rate and concomitant ethanol production range. This analysis was repeated for each of the reaction deletion strains that were shown to result in an increase in ethanol production (Figure 3). Figure 6 explores the predicted maximum ethanol yields when supplementing lactate, malate, or both lactate and malate to either the wild-type or gene deletion strains. Comprehensive results for all 35 tested metabolites are available in Additional file 1. When these metabolites are added to the wildtype strain, there is no increase in the maximum ethanol yield, but in the deletion strains, these additions significantly improve ethanol production. When both lactate and malate are added to the deletion strains, maximum ethanol yield is increased by ~50% for the deletion strains affecting hydrogen production (ΔFDXHASE) or electron transport (ΔNFO) and ~140% for Δpta and Δack, which knock out acetate production. Other media additions increased ethanol production by thermodynamically blocking production of another competing metabolic byproduct, such as acetate, as is seen experimentally in a metabolic shift assay. For example, if acetate is supplied to the model, acetate production is blocked thermodynamically, and the maximum ethanol yield is increased 11 fold. For the purposes of the model, this effect is exactly the same as deleting the genes coding for acetate kinase (R_ACK) or phosphoacetyltransferase (R_PTA2). By combining the implementation of a single gene deletion and alternative media formulations, the maximum ethanol yield at optimal growth can be increased by as much as 35 fold.

## Discussion

Current and future demands for renewable energy sources have spurred research in developing biofuels. One promising route for biofuel production is to use an organism-based bioprocess where cellulose could be converted to biofuel. One of the main challenges to this approach is that there are relatively few cellulolytic organisms capable of biofuel production, and none of these are especially well-characterized at present. Here we have implemented a computational modeling approach to study *C. thermocellum*, an anaerobic thermophile with high biofuel production potential. In this study, the development of a genome-scale metabolic model of *C. thermocellum *was used to provide a framework for analyzing the basic metabolic functions of *C. thermocellum *and improving its ethanol production capabilities. Overall, we report the construction of a genome-scale metabolic model of *C. thermocellum*, *i*SR432, and the accuracy of this model to predict cellular phenotypes (growth and fermentation product secretion) for growth on cellobiose and fructose in continuous and batch culture. Specific results of significance were: 1) the generation of a weighted amino acid representation of a cellulosome based upon proteomic data, 2) suggestions for additional genome annotations and identification of unanswered questions related to metabolism, and 3) identification of a general design principle related to intracellular reduction-oxidation balance that strongly influences the selection of gene deletions and chemical environments to increase ethanol production in *C. thermocellum*.

We have constructed a genome-scale constraint-based model of *C. thermocellum *metabolism. The model accounts for 432 genes and includes 577 reactions involving 525 intracellular metabolites. Some thermodynamic constraints are placed on the metabolic solution space in the form of irreversible reactions. This reconstruction was tested by comparing computational predictions to experimentally measured growth rates and fermentation product secretion fluxes. By including as few as two experimentally determined values (substrate uptake rate and one fermentation-product secretion rate) as model constraints, we find that the model's predicted growth rate closely matches the experimentally observed value for continuous culture growth on cellobiose and fructose [32]. The addition of a third constraint (substrate uptake rate and two fermentation product secretion rates) gave reasonably accurate predictions for both continuous and batch culture growth. We should note that this step of testing the computational predictions to quantitative experimental data was a critical step to finalizing our current model. Prior to this step, our model calculated physiologically reasonable growth rates and by-product secretion profiles; however, there were several futile cycles that remained undetected until there were quantitative values of growth for comparison. The current model has no futile cycles resulting from computational artifacts (see Additional file 4 for details on removed reactions to prevent futile cycling). Comparison of model content from our model with that of models for *C. acetobutylicum *and *S. cerevisiae *reflects the expected relationships between the organisms; i.e., the two Clostridia models show much more overlap with each other than with the *S. cerevisiae *model. Shared functionality tends to be concentrated in central metabolism and nucleic acid metabolism, while primary differences tend to occur in how each organism can process starch and sucrose. Significantly, despite a large overlap between the clostridial models, significant functional differences are manifest between the models based on a relatively small number of reaction discrepancies. This points to the utility of constraint based models for making detailed functional predictions based on genome content, as well as the importance of correct genome annotation.

In the process of constructing the model for *C. thermocellum*, we developed a weighted amino acid representation of a cellulosome that can be included as a component of the cellular objective required for growth. Experimental evidence suggests that the cellulosome, the extracellular structure responsible for cellulose degradation, comprises as much of 20% of the dry weight of *C. thermocellum *in certain conditions [40], and thus the metabolic demands associated with cellulosome production are significant. Cells experience a cost-benefit tradeoff when expressing proteins [41], thus we felt it necessary to formulate a cellulosome-specific component in our model. The current representation suggests that the increased production demand associated with the cellulosome decreases the growth rate by 4-17%. *In vivo *cellulosome production is of course dynamic, however, and the *in silico *representation is therefore designed to be tunable to available experimental data for different conditions by varying the relative contribution of the mass of the cellulosome to biomass or the ATP input required for cellulosome production. To our knowledge, our inclusion of an amino acid-weighted representation of a cell substructure, i.e. the cellulosome, as part of the biomass equation is the first such formulation used in constraint-based models.

One of the useful features of genome-scale constraint-based models is that they can focus attention on areas of metabolism that are relatively unexplored, or illuminate high-priority areas for future research [42]. In light of *i*SR432, two examples of poorly characterized areas of metabolism in *C. thermocellum *that require further investigation were identified during the gap filling phase of model construction. These two areas are: the fate of succinate and the possibility of anaerobic respiration in this organism. Genomic evidence suggests an incomplete citric acid cycle in *C. thermocellum*. In our simulations, we found the citric acid cycle operates in the "forward" direction up through succinate, resulting in production and secretion of succinate. This behavior is not necessarily preferred by our model, unless constraints are placed on the production of other, more energetically favorable fermentation products. Secretion of succinate by *C. thermocellum *has been previously reported [31], although recent research does not show succinate as a major fermentation product of metabolism [32,37]. Thus, it is not clear if the citric acid cycle is complete or if it is incomplete resulting in production of succinate. In the current version of the model, all reactions of the citric acid cycle are present (based upon genome annotations and biochemical evidence) with the exception of succinate dehydrogenase. This question led to an experiment to assay for succinate dehydrogenase activity in *C. thermocellum*, however no activity was found. This leads us to believe that the citric acid cycle in *C. thermocellum *is not complete and there remains an open question regarding the cellular fate of intracellular succinate. If succinate is not secreted by *C. thermocellum*, or is secreted in very small amounts, this indicates that there must be intracellular fates of succinate that are not captured by current genome annotations or our current model. Possibilities include further processing via propanoate, glyoxalate, or tyrosine metabolism; though none of these seems likely on the basis of the available genomic evidence.

Anaerobic respiration is another area of interest highlighted in our studies using *i*SR432. We found genomic evidence for NADH-quinone oxidoreductase. While there is no clear genomic evidence for a nitrate reductase, there is a *C. thermocellum *gene, Cthe_0199, that is a reciprocal best hit for a gene in the prokaryotic molybdopterin-containing oxidoreductase family [43], which includes nitrate reductases. Some, but not all, clostridia species are known to reduce nitrate [44]. There is at least one report specifically stating that *C. thermocellum *does not reduce nitrate under the conditions studied [31]. If *C. thermocellum *does not possess nitrate reductase or some other similar reaction, it is not clear how reduced quinone generated by NADH-quinone oxidoreductases might be reoxidized and it may be possible that the NADH-quinone oxidoreductase may be incorrectly annotated.

One of our goals in constructing a model of *C. thermocellum *was to create a useful tool for strain design [10,22,45], so that interventions to increase the production of ethanol (or other desired fermentation products of interest) could be evaluated or designed *in silico *prior to experimental laboratory work. A simple illustration of this is provided by the results in Figure 3, which shows non-lethal gene deletions expected to increase ethanol production. As expected, gene deletions that inhibit acetate production were found to increase the upper bound on ethanol secretion. We also found that eliminating reactions involved in the recycling of NADH back to NAD, namely NADH:ferredoxin oxidoreductase and ferredoxin hydrogenase, increase the upper bound on ethanol secretion. These results indicated a general relationship between the reduction-oxidation status of *C. thermocellum *and the production of ethanol. This finding was demonstrated more specifically in Figure 4 where the relationship between H_2 _and ethanol production is shown. As H_2 _production increases, ethanol production falls, eventually to zero. These results indicate that the capacity of *C. thermocellum *for ethanol production is strongly influenced by intracellular reduction-oxidation balance. Future strain design work in *C. thermocellum *likely needs to consider this aspect of *C. thermocellum*'s cellular physiology. This finding also points to the utility of using genome-scale metabolic models to facilitate the strain design process by reducing the workload associated with manually accounting for redox considerations.

Additional computational analyses were conducted to study the effects of modifying the chemical environment of *C. thermocellum*. It was found that the addition of lactate or malate to the growth medium of *C. thermocellum *should induce a marked increase in ethanol secretion for a number of gene deletion strains. These results allude to the utility of specifying environmental conditions as a design parameter for engineering strains. In addition, the change in fermentation production secretion profile occurs naturally in *C. thermocellum *over the course of the its growth cycle as fermentation products secreted early in fermentation can influence what fermentation products are secreted in the late stages of fermentation.

## Conclusions

In this study, we applied constraint-based modeling to a genome-scale metabolic reconstruction of the cellulolytic, ethanologenic bacterium *C. thermocellum *in an effort to expedite research on this organism that has a high biofuel production potential. By incorporating genomic sequence data, network topology, and experimental measurements of enzyme activities and metabolite fluxes, we have generated a model that is reasonably accurate at predicting the cellular phenotype of *C. thermocellum *(at least for the environments shown in this study). The *i*SR432 model establishes a foundation for the integration and interpretation of large-scale systems biology data sets because the model's predictions can be further refined through the incorporation of thermodynamic constraints, gene regulatory data, and enzyme kinetics and because resulting models can be used for future strain design work involving combinations of gene modifications and chemical environments. Even at the level of studying single gene deletions in combination with chemical additions, analysis using the *i*SR432 model resulted in an interesting observation that ethanol production is influenced by the reduction-oxidation state of the cell. These observations illustrate the utility of this model as a predictive tool for rational manipulation of *C. thermocellum *metabolism.

## Methods

### Construction of an in silico genome-scale stoichiometric model of *C. thermocellum *metabolism

#### Reaction list

The core of the stoichiometric metabolic model is a list of metabolic reactions occurring in *C. thermocellum*, compiled based on evidence from genome annotations and experimental observations. An initial list of biochemical reactions was assembled based on predicted enzymatic functions in the genomic annotations available from IMG, UniProt, and KEGG [23-27]. Specifically, Enzyme Commission (EC) numbers of annotated *C. thermocellum *genes were used to select reactions from a set of database reactions, assembled using previously published constraint-based metabolic models [1,3,21,46]. Transport reactions, representing the movement of metabolites between the extracellular space and cytosol, were initially added based on the annotations, or based on similarity searches between the *C. thermocellum *genome and the Transport Classification Database (TCDB) [47]. For the latter, we identified reciprocal best hits between *C. thermocellum *genes and genes in the Transport Classification Database (TCDB) using BLASTP with an e-value cutoff of 10-5, and then added reactions according to mechanisms proposed for the genes from TCDB, where available. This initial assembly contained many broken, incomplete, and/or isolated pathways and subsystems. Furthermore, many reactions involving the synthesis of large, complex molecules, e.g., cell wall teichoic acids and extracellular proteins, were missing from this initial reaction list.

To represent the synthesis of large molecules such as phospholipids and cell wall teichoic acid, we created lumped reactions that produced an "average" of that molecular species from a representative fraction of small molecules. For example, to account for the synthesis of 1-acyl-glycerol 3-phosphate, we first created lumped reactions synthesizing necessary fatty acyl-CoA molecules from malonyl-CoA, acetyl-CoA, and other cofactors. Next, we used percent w/w fatty acid content for *C. thermocellum *as determined by Chan et al. [48] and Herrero et al. [49] to construct a reaction synthesizing a representative molecule of 1-acyl-glycerol 3-phosphate. The coefficients for each of the fatty acids in this reaction are based on the fatty acid content of the cell (see calculations in the Additional file 5). The resulting average glycerophospholipids were then available for conversion to other components of the phospholipid bilayer and cell wall. This approach has been used previously [1,2].

#### Definition of biomass flux reaction

In order to analyze a constraint-based model by FBA, one must specify a metabolic objective. We specified maximization of biomass as *C. thermocellum*'s metabolic objective. As in previous work, we represented biomass production by a lumped reaction, with metabolites crucial for biomass formation drained in proportion to their respective requirements [1,50]. The identity of these metabolites and the required amounts for each was determined for *C. thermocellum *using experimental data whenever possible. In the absence of experimental data specific for *C. thermocellum*, we used similar data from the genome scale metabolic model of the gram positive bacterium *Bacillus subtilis *[2]. Although the specific requirements of *B. subtilis *no doubt differ from those of *C. thermocellum*, the results of FBA are relatively insensitive to minor misspecification of biomass requirements [51]. The cellular dry weight was divided into the broad categories of protein, RNA, DNA, lipids and cell wall, and ions and metabolites, and each category was divided into subcategories to obtain the molar contribution of individual metabolites to cell mass. Distribution of individual amino acids, deoxyribonucleic acids, and ribonucleic acids in the protein, DNA, and RNA categories was determined by performing counts of each in either the whole genome (for DNA) or in every open reading frame (for RNA and protein). See Additional file 5 for detailed calculations and results.

#### Gene-protein-reaction (GPR) relationships

GPR relationships specify the putative relationship between genes and enzymatic activities in an organism. Following previous work [52], we represented these relationships as Boolean statements. The simplest such statement was: gene A implies reaction X, i.e., gene A -> reaction X. Enzymatic activities associated with protein complexes required more complicated statements, e.g., (gene A or gene B) and (gene C) -> reaction X.

To develop the GPR relationships for *C. thermocellum*, we used the annotations described above and information on protein complexes from UniProt. For each EC number in the *C. thermocellum *annotations, we searched UniProt [23] to determine whether that EC is associated with protein complexes, and if so, what type of complex exists across different organisms (homodimer, heterotrimer, etc.). Based on this information, and the information in the annotations, we assigned putative GPR relationships for *C. thermocellum*, conforming to known enzyme complex architecture whenever possible. As a specific example of this process, consider the reaction corresponding to carbamoyl-phosphate synthase ('R_CBPS'), EC 6.3.5.5. Several UniProt entries (e.g., CARA_ECOLI) with this EC number are annotated as being members of a complex composed of two chains, a small glutamine-hydrolyzing chain and a large chain that synthesizes carbamoyl phosphate. We found two *C. thermocellum *genes annotated as "carbamoyl-phosphate synthase, small subunit" (Cthe_1867, Cthe_0950) and two genes annotated as "carbamoyl-phosphate synthase, large subunit" (Cthe_1868, Cthe_0949). Thus, we expressed the GPR relationship for this reaction as: (Cthe_1867 or Cthe_0950) and (Cthe_1868 or Cthe_0949) -> R_CBPS.

#### Accounting for cellulosome production

*C. thermocellum *is one of a number of cellulolytic microorganisms that breaks down cellulose via a large (from 2 to 16,000 kDa), multi-functional polypeptide assembly of extracellular enzymes and scaffold proteins called the cellulosome [53]. The amount of cellulosome produced by *C. thermocellum *varies considerably across specific conditions. For example, the amount was found to be ninefold greater in Avicel-grown batch cultures compared to cellobiose-grown batch cultures [40]. Cellulosome-associated polypeptides can be present in quantities as high as 20% percent of the cell mass exclusive of cellulosomes [40], and this likely places a significant additional metabolic burden on the cell. To address this issue, *i*SR432 includes a means of including an additional "cellulosome" requirement in the biomass objective. A lumped reaction was created to represent the formation/export of the cellulosome from the constituitive amino acids and using ATP. The pseudometabolite "cellulosome," representing the outcome of this reaction, can be optionally added to the "cellmass" fraction of biomass discussed above to form total biomass. The molar coefficients of each amino acid to form a single "mole" of cellulosome were determined based on a proteomics study of the *C. thermocellum *cellulosome, which produced the relative abundances of individual peptide constituents of the cellulosome [54]. These data were combined with the protein sequences of each peptide to determine the relative molar abundance of amino acids present in the cellulosome, where one mole of cellulosome equals the average molecular weight of one of its amino acid residues. The ATP cost of assembly as well as the proportion of cellulosome to cellmass needed to create biomass can both be adjusted either directly based on calculated anabolic costs or measured cellulosome production or indirectly to match observed experimental growth patterns. For the purpose of this study, it was assumed that one ATP molecule must be hydrolyzed to ADP and inorganic phosphate to build and export a single average cellulosome building block. The complete external cellulosome, when expressed, was taken to be present at 20% of the total cell mass [40].

#### Model naming convention

We followed the previously established convention [55] for naming genome-scale constraint-based models, i.e., "*i*" to denote "*in silico*," followed by the initials of the first author ("SR"), followed by the number of genes included in the model ("432").

#### Cell growth

*C. thermocellum *(ATCC 27405) was cultured anaerobically at 55°C in serum bottles sealed with butyl rubber stoppers and purged using ultra high purity nitrogen gas. Standard growth medium for *C. thermocellum *contained in final concentrations 5 g/L cellobiose, 3 g/L sodium citrate tribasic dihydrate, 1.3 g/L ammonium sulfate, 1.43 g/L potassium phosphate monobasic, 1.8 g/L potassium phosphate dibasic, 0.13 g/L calcium chloride dihydrate, 6 g/L glycerol-2-phosphate disodium, 4.5 g/L yeast extract, 2.6 g/L magnesium chloride hexahydrate, 1.1 mg/L ferrous sulfate heptahydrate, 0.5 g/L cystein-HCl, 50 mM MOPS buffer, and 0.1% resazurin. *Escherichia coli *was cultured aerobically at 37°C in M9 minimal growth medium containing 2 g/L glucose.

#### Assay for succinate dehydrogenase (SDH) and lactate dehydrogenase (LDH) enzymatic activity

The enzymatic activity of succinate dehydrogenase in *C. thermocellum *was tested using a modification of the method used by Kun and Abood [56] in which a tetrazolium dye is reduced by the dehydrogenase enzyme in the conversion of succinate to fumarate. This assay was also used to detect the activity of lactate dehydrogenase by substituting lactate for succinate in the assay. Cells were harvested in late log phase, centrifuged for 15 min at 8000 g, washed once and resuspended in half the original volume of distilled water. Into a 15 mL centrifuge tube were added 0.5 mL of phosphate buffer at pH 7.8, 0.5 ml of either succinate or lactate at 0.2 M concentration, 1.0 mL of either 100% or 50% cell suspension, and 1.0 mL of 0.1% nitro blue tetrazolium. The tubes were incubated in either a 37°C (*E. coli*) or 50°C (*C. thermocellum*) water bath for 20 minutes. Tubes were removed, and the reaction was stopped by adding 7 mL acetone and shaking well. A blue color indicated reduction of the tetrazolium dye, and thus, the presence of the query enzyme activity.

### Model analysis

#### Flux Balance Analysis

Once constructed, we analyzed the *C. thermocellum *genome-scale constraint-based model using FBA [4]. In essence, FBA uses linear programming to identify a single point in the space of possible steady state metabolims that optimizes a given metabolic objective. As discussed above, we specified maximization of flux on the biomass reaction as the metabolic objective. The linear programming problem of FBA can be expressed as

Maximize: Z

Subject to: *S *• *v *= 0,

*a*_*i *_<*v*_*i *_<*b*_*i *_for all reactions i,

where Z is the flux on the biomass reaction, S is the stoichiometric matrix (discussed below), and ***v ***is the vector representing the flux values of each reaction in the model (the flux distribution) [4]. The first statement gives the objective. The second statement encapsulates the steady-state assumption, that the concentrations of all metabolites are unchanging. The third statement encapsulates specific flux constraints for each reaction, e.g., whether the reaction is reversible, or not (if the reaction is irreversible, then the lower bound is set to zero). These bounds can also be used to constrain individual reactions to zero flux (a_*i *_= b_*i *_= 0) or other experimentally-determined flux values. If no information was available, the upper and lower flux bounds were set to 1000 and -1000 mmol gDW^-1 ^hr^-1^, respectively. The stoichiometric matrix S is the mathematical representation of the reaction list. It has one row for each metabolite and one column for each reaction. Each element of S, s_*ij*_, represents the stoichiometric coefficient of the *i*th metabolite in the *j*th reaction (the coefficients are positive when the metabolite is a product of the given reaction and negative when the metabolite is a reactant). The vector ***v ***contains the flux values for all reactions in the model, i.e., v_*i *_is the flux on the *i*th reaction.

#### Essentiality

Constraint-based models can be analyzed by FBA to make comprehensive *in silico *gene essentiality predictions [2]. For each gene in the model, that gene is assumed to be deleted or nonfunctional. Then, using the GPR relationships, the effect of the *in silico *gene knockout on reaction activities is assessed. If the gene is crucial to the activity of a reaction in the model, then that reaction is constrained to have zero flux to simulate the effect of the gene deletion. Finally, the model with this new constraint is analyzed by FBA, maximizing the growth objective for batch growth on cellobiose. If the maximum flux on the biomass reaction is zero, then the deleted gene is predicted to be essential. If the maximum flux on the biomass reaction is greater than zero, the deleted gene is predicted to be nonessential. All constraints are reset to their default values, and the process is repeated for the next gene in the model.

#### Flux Variability Analysis

FBA is guaranteed to produce an optimal solution to the linear programming problem stated above. However, there generally are many optimal solutions, i.e., different flux distributions that give the same optimal objective value [34,57,58]. In order to examine production capabilities for ethanol, we used flux variability analysis [59] to account for possible variation in ethanol production across optimal solutions. In flux variability analysis, the model is first analyzed by FBA, thus determining the optimal value for the biomass reaction flux. This flux is then imposed as a constraint, i.e., the model is forced to operate at the optimal level. For the present study, we constrained the biomass flux lower and upper bounds to 99% and 100% of the value from FBA, respectively. Then, the flux on a reaction of interest (e.g., ethanol production) is maximized and then minimized by FBA, to find upper and lower bounds on that reaction flux that are consistent with optimal biomass reaction flux.

#### Special Techniques for Model-Model Comparison

We compared genome-scale constraint based models for three key organisms in ethanol production, *C. acetobutylicum *(represented by two models: Cac1 [19,20] and Cac2 [21]) *C. thermocellum *(Cth), and *S. cerevisiae *(Sce) [3]. Direct comparison of model reactions is hampered by the fact that different models use different conventions for naming metabolites and writing reactions. As an example, consider the reaction for hexokinase. In the *C. thermocellum *and *S. cerevisiae *models, this reaction is written as,

atp + glc-D --> adp + g6p + h

while in the Cac1 model, this reaction is written as,

ATP + beta-D-Glucose < = > ADP + beta-D-Glucose 6-phosphate

and in the Cac2 model, the reaction is written,

bDG6P + ADP < -> ATP + bDGLC.

Aside from the different metabolite identifiers, reactions in the Cac1 and Cac2 models are written using the "biochemical" convention, as opposed to the charge-balanced "chemical reactions" used in the others. Usually, this difference is manifested in whether hydrogen is represented as a distinct metabolite in a reaction or not (see above). However, it also results in other differences, e.g., the metabolite NH4 is used instead of NH3 in the *C. thermocellum *and *S. cerevisiae *models, while the opposite is true in the Cac1 and Cac2 models. In addition, the Cac1 and Cac2 models distinguish between different metabolite anomers (e.g., alpha-D-glucose and beta-D-glucose) while the others do not. For these reasons and others, we decided to use EC numbers to compare the contents of each model. EC numbers have the advantage that they are relatively stable over time, represent international consensus on biochemical reaction categorization, and can overcome ambiguities regarding how to represent a given metabolic reaction in a constraint-based model.

Conventions in assigning EC numbers to reactions can vary. In the Cac1 and Cth models, a single reaction could be associated with multiple EC numbers while only one EC number was assigned to each reaction in the Cac2 and Sce models. To ensure that the comparison was uniform, the lists of EC numbers used to represent Cac1 and Cth were compared with the other models, and EC numbers unique to these models (Cac1 and Cth) were only kept if no other EC number for that same reaction occurred in the other models.

There are also no universally adhered to guidelines for assigning reactions (and thus their associated EC numbers) to subsystems. To minimize the impact of this on our EC based model comparisons, we attempted to use KEGG as an "external standard." Specifically, for each EC number encountered in any of the models, we assigned all pathways associated to that EC by KEGG.

This EC-based method of comparison necessarily excludes reactions not linked to an EC number. This means that significant numbers of reactions from subsystems such as extracellular transport and cell envelope biosynthesis are not a part of the comparison. However, the EC-based comparisons do cover most reactions in all four models. Excluding transport reactions, the percentages of reactions not associated with an EC number are 8%, 17%, 12%, ~5%, for the Cth, Sce, Cac1, Cac2 models, respectively.

#### Simulation of alternative media formulations

*iSR432 *was used to simulate the addition of an arbitrary amount of various common metabolites in order to determine the effect on ethanol productivity and yield during batch growth on cellobiose. To simulate an added metabolite, an exchange flux for that metabolite was allowed with the constraints -20 mmol gDW^-1 ^hr^-1 ^≤ *v *≤ 0 mmol gDW^-1 ^hr^-1^. This reflects the thermodynamic prevention of any production of that metabolite and allows the use of it as a source within a reasonable range. For each alternative media formulation, FVA was performed for the ethanol exchange flux and the maximum ethanol flux was used along with the predicted maximum growth rate to calculate the ethanol:biomass yield.

## Authors' contributions

SBR constructed the metabolic model, conducted computational analyses, and drafted the manuscript. CMG participated in model construction and computational analyses. JPB participated in model construction and developed computational algorithms for model construction and analysis. SSF conceived of the study and participated in the design and coordination. All authors read and approved the final manuscript.

## Supplementary Material

Additional file 1 **Model details and results**. This Excel workbook contains the final reaction list, metabolite definitions, biomass function, cellulosome reaction formulation, and gene-protein-reaction (GPR) relationships. In addition, it contains the complete list of results for the reciprocal best hits (RBH) of EC numbers missing from the current C. thermocellum annotations, and the complete results from the single gene- and reaction-deletion simulations and the model comparisons to growth on cellobiose and fructose. Click here for file

Additional file 2 This model file includes tab delimited GPR relationships, reaction list, and sources and escapes for growth on minimal media. Click here for file

Additional file 3 This file contains the model in SBML format. Click here for file

Additional file 4 **Removed futile cycles**. This results file shows reactions which were deleted from the original model build to remove futile cycles. Click here for file

Additional file 5 **Calculations and additional methods**. This file contains detailed methods and sample calculations for determining the biomass objective reaction and the fatty acid content reactions as well as details about simulations of alternative media formulations. Click here for file
