# Supplementary material for: Genome-scale metabolic analysis of Clostridium thermocellum for bioethanol production
Source: BMC Syst Biol. 2010 Mar 22;4:31. doi: 10.1186/1752-0509-4-31 (PMC2852388; doi:10.1186/1752-0509-4-31)
Supplement: Additional file 5 — Calculations and additional methods. This file contains detailed methods and sample calculations for determining the biomass objective reaction and the fatty acid content reactions as well as details about simulations of alternative media formulations. [file 1752-0509-4-31-S5.DOC]

Biomass calculations
A theoretical biomass reaction is generally accepted as a suitable objective function for predicting behavior in many growth conditions. This reaction necessitates the specification of all molar constituents of a "mole" of biomass, defined to be 1 g dry cell weight, along with an ATP maintenance cost. To calculate the molar composition of biomass, we followed well established procedures. Where possible, data specific to C. thermocellum was used, but where necessary we used information specific for the gram positive microorganism Bacillus subtilis, for which there already exists a genome scale metabolic model (Oh et al 2007). All calculations for the coefficients of the biomass reaction are included in the supplementary spreadsheet "cth_biomass.xls." As an example, we present here the calculation of the coefficient for the amino acid alanine. Protein was found to make up 52.84 %w/w of B. subtilis, and the distribution of amino acids within that category was determined using a count of the open reading frames of C. thermocellum. Alanine was counted 68,886 times out of a total of 1,079,799 amino acid residues, giving a percent prevalence (assuming uniform translation of all ORF's) of 6.38% or 0.0638 moles Ala / moles protein. The contribution of Ala to total protein weight was determined using the calculation

(0.0638 moles Ala / moles prot ) * (89.05 g Ala / moles Ala) = ( 5.6810 g Ala / moles prot )

The individual weight contributions of each amino acid were determined likewise and the sum represented the molar mass of total protein. Percent-weight contributions of each amino acid can then be calculated thus:

(5.6810 g Ala / moles prot ) / (130.6664 g prot / moles prot) = (0.0435 g Ala / g prot)

The biomass reaction coefficient is then calculated using the cellular composition of protein:

(0.0435 g Ala / g prot) * (0.5284 g prot / g DW) * (1000 mmol Ala / mol Ala) / (89.05 g Ala / mol Ala) = (0.2580 mmol Ala / g DW)

This coefficient is used for alanine directly in the biomass reaction.

Fatty acid content calculations
The variety and distribution of fatty acid chains in the membrane of C. thermocellum are accounted for by using lumped reactions for the biosynthesis of each type of fatty acyl-CoA as shown here for the synthesis of palmitate and subsequently palmitoyl-CoA:

Ketoacyl-synthase - palmitate:
acetyl-CoA + 20.0 H+ + 7.0 malonyl-CoA + 14.0 NADPH --> 7.0 CO2 + 8.0 CoA + 6.0 H20 + palmitate + 14.0 NADP

Fatty acid - CoA Ligase:
ATP + CoA + palmitate <==> AMP + palmitoyl-CoA + ppi

All fatty acid types are then incorporated together as a single "average" fatty acyl chain during the synthesis of mono-, di-, and triacyl-glycerols which are then manipulated to form other cell envelope constituents:

Glycerol 3-phosphate acyltransferase:
glycerol 3-phosphate + 0.051 decanoyl-CoA + 0.022 tridecanoyl-CoA + 0.023 iso-tetradecanoyl-CoA + 0.0092 tetradecanoyl-CoA + 0.022 tetradecenoyl-CoA + 0.323 iso-hexadecanoyl-CoA + 0.2293 palmitoyl-CoA + 0.1334 anteiso-heptadecanoyl-CoA + 0.1255 hexadecenoyl-CoA + 0.0479 iso-stearoyl-CoA + 0.0137 stearoyl-CoA --> 1.0 1-Acyl-sn-glycerol 3-phosphate + CoA

The calculations used to compile this reaction are shown in the supplementary spreadsheet "cth_biomass.xls"

Alternative Media Simulations

*Clostridium thermocellum iSR432* was used to simulate the growth of wild type and deletion strains with cellobiose and additional supplementary metabolites. Metabolites from the following table were added individually. Media supplementation was simulated by allowing the associated exchange reaction to vary between -20 and 0, thereby allowing a reasonable uptake value and simulating osmotic inhibition of the secretion of the metabolite.

| R_EXCH_ac_e | Acetate |
| --- | --- |
| R_EXCH_cit_e | Citrate |
| R_EXCH_mal_DASH_l_e | Malate |
| R_EXCH_akg_e | α-keto glutarate |
| R_EXCH_for_e | Formate |
| R_EXCH_fum_e | Fumarate |
| R_EXCH_fru_e | Fructose |
| R_EXCH_lac_DASH_l_e | Lactose |
| R_EXCH_rib_DASH_d_e | Ribose |
| R_EXCH_xyl_DASH_d_e | Xylose |
| R_EXCH_glyc_e | Glycerol |
| R_EXCH_cys_DASH_l_e | Cysteine |
| R_EXCH_met_DASH_l_e | Methionine |
| R_EXCH_pro_DASH_l_e | Proline |
| R_EXCH_thr_DASH_l_e | Threonine |
| R_EXCH_asn_DASH_l_e | Asparagine |
| R_EXCH_his_DASH_l_e | Histidine |
| R_EXCH_trp_DASH_l_e | Tryptophan |
| R_EXCH_gly_e | Glycine |
| R_EXCH_lys_DASH_l_e | Lysine |
| R_EXCH_succ_e | Succinate |
| R_EXCH_leu_DASH_l_e | Leucine |
| R_EXCH_ile_DASH_l_e | Isoleucine |
| R_EXCH_arg_DASH_l_e | Arginine |
| R_EXCH_phe_DASH_l_e | Phenylalanine |
| R_EXCH_val_DASH_l_e | Valine |
| R_EXCH_tyr_DASH_l_e | Tyrosine |
| R_EXCH_gln_DASH_l_e | Glutamine |
| R_EXCH_ser_DASH_l_e | Serine |
| R_EXCH_ala_DASH_l_e | Alanine |
| R_EXCH_asp_DASH_l_e | Asparagine |
| R_EXCH_glu_DASH_l_e | Glutamate |

The following figure provides further details that were used for the calculation of the ethanol yield in Figure 6. The box indicates the range of possible ethanol production, and the x indicates the associated growth rate, seen on the secondary axis. The upper bound for ethanol production and the growth rate were used to calculate the maximum ethanol yield per biomass.

References

Oh Y, Palsson B O, Park S M, Schilling C H, and Mahadevan R. 2007. Genome-scale Reconstruction of Metabolic Network in Bacillus subtilis Based on High-throughput Phenotyping and Gene Essentiality Data. *J. Biol. Chem.* 282: 28791-28799.
